# TARNAS: A Software Tool for Abstracting and Translating RNA Secondary Structures

**DOI:** 10.3390/ijms26125728

**Published:** 2025-06-15

**Authors:** Michela Quadrini, Piero Hierro Canchari, Piermichele Rosati, Luca Tesei

**Affiliations:** School of Sciences and Technology, University of Camerino, Via Madonna delle Carceri 7, 62032 Camerino, Italy; michela.quadrini@unicam.it (M.Q.); piero.hierrocanchari@studenti.unicam.it (P.H.C.); piermichele.rosati@studenti.unicam.it (P.R.)

**Keywords:** RNA secondary structure formats, abstractions of RNA secondary structures, statistics on RNA secondary structures, RNAML

## Abstract

Ribonucleic acids (RNAs) fold into complex structures that are strongly associated with their biological functions. These can be abstracted into secondary structures, represented as nucleotide sequences annotated with base-pairing information. This abstraction is both biologically relevant and computationally manageable. Comparing and classifying RNA molecules typically relies on these secondary structure representations, which exist in multiple formats. In this work, we introduce TARNAS 1.0, a software tool designed to convert RNA secondary structure representations across multiple formats, including Base Pair Sequence (BPSEQ), Connect Table (CT), dot-bracket, Arc-Annotated Sequence (AAS), Fast-All (FASTA), and RNA Markup Language (RNAML). The tool offers options for retaining or removing comments, blank lines, and headers during the conversion process. These format translation and preprocessing capabilities are specifically designed to support the batch handling of large collections of RNA molecules, making TARNAS well suited for large dataset construction and database curation. Beyond format translation, TARNAS computes three levels of abstraction for RNA secondary structures, namely core, core plus, and shape, as well as a set of statistical descriptors for both primary and secondary structure. These abstraction and analysis features are intended to facilitate the comparison of molecules and the identification of recurring structural patterns, which are essential steps for associating structural motifs with molecular function. TARNAS is available as both a standalone desktop application and a web-based tool. The desktop version supports batch processing of large datasets, while the web version is optimized for the analysis of single molecules.

## 1. Introduction

Ribonucleic acids (RNAs) are single-stranded molecules that encode genetic information and perform biological functions, including transcription, splicing, translation, and regulation of protein function. Each single strand consists of four nucleotides: adenine (A), guanine (G), cytosine (C), and uracil (U). Such single-stranded molecules fold onto themselves, resulting in complex three-dimensional configurations. Different base pairs are established between nucleotides during the folding process. These are commonly classified as canonical, such as Watson-Crick-Franklin base pairs (A–U and G–C), and non-canonical, which include wobble (G–U), sheared, and Hoogsteen interactions [1]. Although G–U wobble pairs are sometimes considered non-canonical, they are frequently observed in RNA stems and exhibit a geometry closely resembling canonical base pairs.

**Figure 1:**
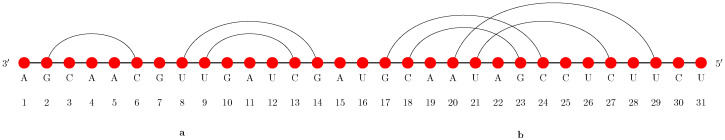
Arc diagram representation of RNA secondary structures. Red circles indicate nucleotides, while arcs denote base-pair interactions. The motif shown in part (**a**) is pseudoknot-free, whereas the one in part (**b**) contains a pseudoknot.

Secondary structures are molecular abstractions that disregard molecular spatial configuration and consider only canonical base pairs. Each secondary structure can be schematically represented as an arc diagram, as shown in Figure 1, where the vertices represent nucleotides in a straight line (backbone), and the base pairs are drawn as arcs in the upper half-plane. RNA secondary structures may be pseudoknot-free or include pseudoknots. A structure is pseudoknot-free if its corresponding arc diagram exhibits no crossing interactions between base pairs (Figure 1a). Conversely, it is classified as pseudoknotted when such crossings are present (Figure 1b).

Analyzing RNA secondary structures is essential for understanding RNA function across a wide range of biological contexts, as this abstraction retains biologically meaningful features while remaining computationally tractable. The comparison and classification of RNA secondary structures are critical in applications such as function prediction and the study of regulatory mechanisms in gene expression. To assess structural similarity or dissimilarity, a variety of alignment-based techniques have been developed. For instance, RNAdistance [2] and PSMAlign [3] use edit-distance-based approaches, while RNAforester [4,5] applies tree alignment methods. Other tools, including MiGaL [6], TreeMatching [7], Gardenia [8], and ASPRAlign [9], follow similar alignment strategies. Additionally, approaches such as PskOrder [10] and RAG-2D [11] incorporate concepts from graph theory and topological analysis.

These tools often rely on different input formats for RNA secondary structures due to the absence of a universal standard. For instance, PSMAlign requires structures in dot-bracket format, whereas RAG-2D uses the Connect Table (CT) format. Moreover, some tools, such as ASPRAlign, support multiple formats, including Base Pair Sequences (BPSEQ) and Arc-Annotated Sequences (AAS). Finally, public databases do not always provide RNA secondary structures in all formats. Consequently, structure conversion is usually needed when combining data from multiple sources or using different tools in parallel.

Several tools have been proposed in the literature to convert RNA secondary structures across different formats, including RNApdbee [12] and its later versions, RNApdbee 2.0 and 3.0 [13]. These web-based platforms provide useful functionalities such as the derivation of 2D structures from 3D data and visualization features. However, they are limited to processing a single RNA structure at a time, which restricts their applicability for tasks such as benchmark construction, dataset generation, and large-scale comparison or statistical analysis. For example, in [14], some of the authors proposed a framework to evaluate RNA secondary structure comparison methods, both with and without pseudoknots. Building a comprehensive dataset to assess the various tools was time-consuming and error-prone, emphasizing the need for a scalable solution capable of handling multiple formats and large sets of RNA molecules.

In this work, we present TARNAS, preliminarily introduced in [15], a software tool designed to translate RNA secondary structure formats in single and batch modes. TARNAS supports the main formats for encoding RNA secondary structures, including BPSEQ, CT, dot-bracket, and Arc-Annotated Sequences (AAS), representing Watson–Crick–Franklin base pairs and wobble pairs. In addition, TARNAS supports FASTA, which encodes only the primary RNA sequence without structural information, and RNAML, an XML-based format capable of representing not only secondary structures, but also non-canonical interactions and tertiary structure annotations. These formats, except RNAML, are typically encoded as plain text files consisting of two main sections: a header containing metadata such as the organism name or ID code and a body describing the structural or bonding information of the molecule. Section 3.1 describes each of the supported formats in detail.

**Figure 2:**
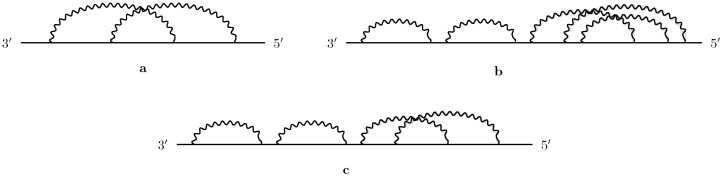
The shape (**a**), core (**b**), and core plus (**c**) abstractions of the RNA secondary structure depicted in Figure 1.

In addition to format translation, TARNAS provides advanced capabilities for the structural abstraction and analysis of RNA secondary structures. It implements three levels of abstraction—core, core plus, and shape—which reduce structural complexity while preserving essential features. Shapes, originally introduced in [16,17], are obtained by removing unpaired nucleotides and collapsing parallel arcs into a single arc. Two arcs are considered parallel if they are nested and no nucleotides lie between their endpoints. The core and core plus abstractions, proposed by the authors in [15], follow the same general principle but differ in the order of operations: in core, unpaired nucleotides are removed first, followed by arc collapsing; in core plus, arcs are collapsed first, and unpaired nucleotides are then removed. Figure 2a–c illustrates the core, core plus, and shape abstractions of the RNA molecule shown in Figure 1. Complementing these abstractions, TARNAS also offers several analytical and preprocessing options, such as generating statistics files, choosing to include or exclude non-canonical base pairs, and retaining or discarding headers during format translation. These abstraction and analysis functionalities are designed to support large-scale comparison tasks and the identification of conserved structural patterns, which are essential for linking RNA structure to molecular function.

The remainder of this paper is organized as follows. Section 2.1 presents the architecture of TARNAS along with the available versions. Section 2.2 describes the user interface and the format translation functionalities. Section 2.3 illustrates the application of structural abstractions on a large dataset, while Section 2.4 explores the use of statistical descriptors. Section 3.1 provides a detailed overview of the supported formats, and Section 3.2 explains the abstractions that can be computed. Finally, Section 4 summarizes the contributions of this work and outlines directions for future research.

## 2. Results and Discussion

This section presents the architecture and distribution of TARNAS, followed by a detailed demonstration of its functionalities on a large dataset.

### 2.1. TARNAS Architecture and Distribution

The TARNAS tool is implemented in Java and uses the ANTLR4 tool [18] to generate parsers from grammars that formally define the syntax of each supported format. RNAML, due to its XML-based structure, is instead processed using the W3C Document Object Model (DOM) API [19]. The source code and version 1.0 release of TARNAS are available at [20], while a companion tutorial—including the dataset used in this work and the ANTLR4 grammar definition—is provided in [21].

TARNAS is distributed in two versions: a standalone desktop application and a web-based application. The desktop version, available at [20] as standalone executable JAR files, supports batch processing for efficient handling of large datasets. It is compatible with Linux, Windows, and macOS systems running Java SE Runtime Environment 19 or higher. Both a graphical user interface (GUI) and a command-line interface (CLI) are provided, allowing users to choose between interactive use and integration into automated computational pipelines. The CLI version of TARNAS can be called from the command line as follows:

> java -jar TARNAS_CLI.jar [options]
Option -h displays a complete list of available commands and options.

The web application, accessible at https://bdslab.unicam.it/tarnas/ (accessed on 14 May 2025), is built using Spring Boot [22] and Node.js [23] on the back end, and Angular [24] for the single-page front end. Unlike the desktop version, the web interface is currently limited to processing one molecule at a time.

TARNAS provides several key functionalities, including format translation and preprocessing, structural abstraction, and the computation of statistical descriptors. These features are described in detail in the following sections.

### 2.2. Translation of Formats

**Figure 3:**
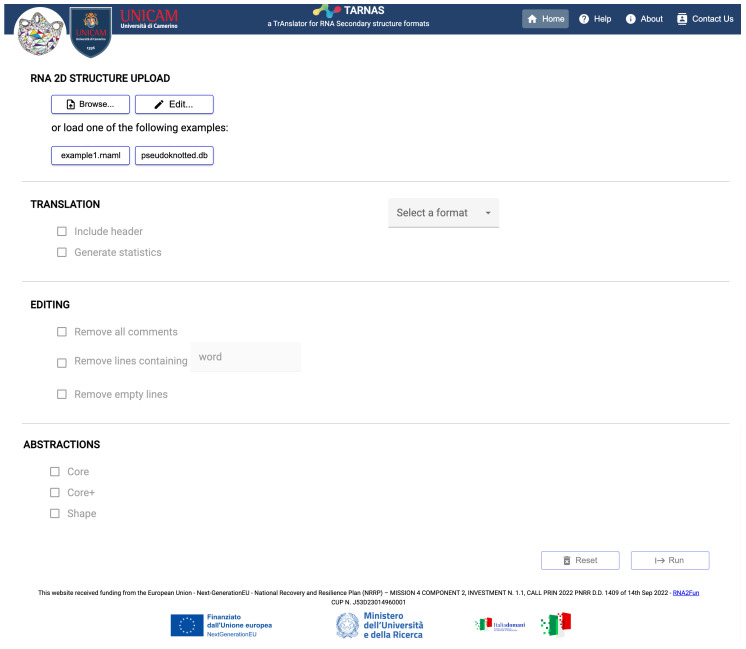
Interface of the TARNAS web app.

**Figure 4:**
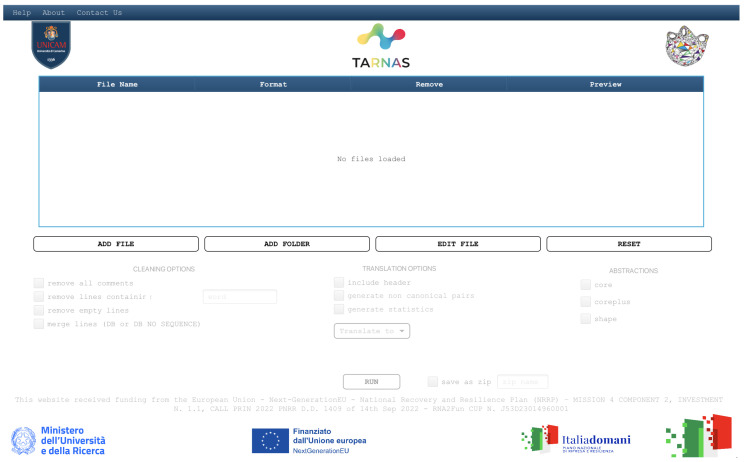
Interface of the TARNAS standalone version.

On the TARNAS home page, which is available in both the web application and standalone GUI versions, users can upload and edit RNA secondary structure files in one of the supported formats: BPSEQ, CT, dot-bracket, AAS, FASTA, and RNAML. The web application processes one file at a time, whereas the standalone application can simultaneously load and process multiple files. Figure 3 and Figure 4 show the respective home pages.

Users can convert uploaded RNA secondary structures into any of the formats supported by TARNAS, as listed in Table 1. Additionally, they can choose whether to include or exclude headers and comments, and optionally generate a statistical summary of the structure.

Tools such as RNAView [25], which extracts secondary structures from the RCSB Protein Data Bank (PDB) [26], may include both canonical and non-canonical base pairs in their output files (e.g., RNAML or other text files). When TARNAS receives an RNAML input file, users can choose whether to retain or discard non-canonical base pairs. If retained, non-canonical pairs (e.g., C–C or G–G) are saved in a separate CSV file because these pairs are not supported by most formats (BPSEQ, CT, dot-bracket, AAS, and FASTA).

### 2.3. Structural Abstractions

We selected a dataset of 1460 5S ribosomal RNA (5S rRNA) molecules that entered the RCSB Protein Data Bank repository [26], containing experimental data on their three-dimensional structure. The secondary structure was extracted using the RNAView tool [25] to produce one RNAML file for each molecule.

Using TARNAS, all molecules in the dataset were translated into the BPSEQ, CT, dot-bracket, AAS, FASTA, and RNAML formats. Subsequently, we computed the core, core plus, and shape abstractions for each molecule. The complete dataset, along with all computed abstractions and the additional data used in the examples, is available in [21].

We now present an example of pattern-based comparison and classification using the shape abstractions. Table 2 reports the number of occurrences for each shape found in the dataset. Notably, a significant portion of the molecules— Group 1, comprising nearly 50%—shared the same shape, which corresponds to a pseudoknot of order 1, commonly referred to as H-type pseudoknot [16,27]. Another large subset—Group 2, approximately 45%—included molecules without a defined shape, typically due to the absence of pseudoknots. Group 3, accounting for only 2.5% of the dataset, consisted of molecules in which an H-type pseudoknot is nested within another H-type pseudoknot. The remaining molecules were aggregated into a final group, as their corresponding shape sets contained only singletons or rare patterns, which were considered statistically insignificant.

Table 3 lists the same computation for the core abstraction. In this case, we observed that the larger groups could be divided into molecules without pseudoknots (2c, 4c, 5c, and 6c, for a total of 623 molecules) and molecules with pseudoknots (1c, 3c, and 7c, for a total of 637 molecules). Molecules without pseudoknots nearly correspond to the null shaped group 2s, which amounts to 667 molecules. Molecules with pseudoknots in groups 1c and 3c (606 molecules) could be abstracted into the major shape group (1s, 717 molecules). The 31 molecules in group 7c can be abstracted into group 3s shapes with 41 occurrences. As in the previous case, the remaining groups contained only a few occurrences and were considered insignificant in this experiment.

Finally, Table 4 presents the computations for the core plus motifs. The less significant groups were far more numerous, accounting for approximately 56% of the molecules. Nevertheless, the first nine groups followed the same pattern as the core, since the molecules without pseudoknots (groups 4cp, 5cp, 6cp, 7cp, and 9cp) correspond to the core without pseudoknots, which in turn correspond to the null shapes (group 2s). Molecules with pseudoknots in groups 1cp, 2cp, and 3cp can be abstracted into core groups 1c and 3c, which are represented by the core group 1s. Similarly, molecules with pseudoknots in group 8cp can be abstracted into core group 7c, which can be further abstracted into shape group 3s.

In summary, the 5S rRNAs in the analyzed dataset can be grouped into three distinct clusters, corresponding to the first three identified shape categories:

1. molecules following the motif of an H-type pseudoknot,
2. molecules without pseudoknots, and
3. molecules featuring an H-type pseudoknot nested within a larger H-type pseudoknot.

This clustering was further corroborated by the analysis of core and core plus abstractions, which reflected similar structural distinctions.

These results are partly expected, given the nature of the abstractions and the well-characterized structure of 5S rRNA. Nonetheless, this example illustrates the potential of TARNAS for structure-based pattern analysis and classification. While the clusters observed here align with known structural properties, applying the same methodology to less studied or heterogeneous datasets could uncover previously undetected patterns and biologically meaningful structural groupings.

### 2.4. Statistics on Sequence and Structural Information

The statistics file currently generated by TARNAS includes the following:

- sequence length and nucleotide counts (for the primary sequence),
- number of base pairs and counts of specific pairs (G–C, A–U, and G–U).

Table 5 shows the mean and standard deviation of the occurrence of nucleotides and bonds in the 1460 molecules of the dataset. These values were obtained from the raw data computed using TARNAS for each molecule, available at [21].

This statistical information can be used for the classification and comparison of primary and secondary structures. For instance, computed statistics and measures can characterize a family of RNAs or a group of functionally similar molecules with a fair degree of precision. This might be a first step toward a subsequent, more tailored investigation.

Moreover, these data can be used to study correlations between primary and secondary structures or a particular abstraction such as core, core plus, and shape. As the structure is related to the function of the molecule, it may provide an opportunity to characterize biological information using a relatively simple and fast computational approach.

## 3. Materials and Methods

Various data formats have been developed to encode RNA secondary structure information. Section 3.1 presents these formats in detail, and Section 3.2 introduces the RNA abstractions that TARNAS can compute.

### 3.1. Data Formats and Translation

BPSEQ [28] is a text format in which each line corresponds to a nucleotide in the primary RNA sequence. Each line includes the nucleotide position (starting from 1 for the leftmost position, 5’), nucleotide base (e.g., A, C, G, U, or other IUPAC code [29] characters), and the position of the paired base. A value of 0 indicates that the nucleotide is unpaired (referred to as a residue).

CT [30] is a text format in which the first line typically contains metadata of a sequence. The standard format for this line is <sequence_length> <sequence_name or description>. Each subsequent line represents a nucleotide and provides the following information: index *i*, the nucleotide at position *i*, 5’-connected base index (i−1), 3’-connected base index (i+1), paired base index (or 0 if unpaired), and original sequence index.

**Figure 5:**
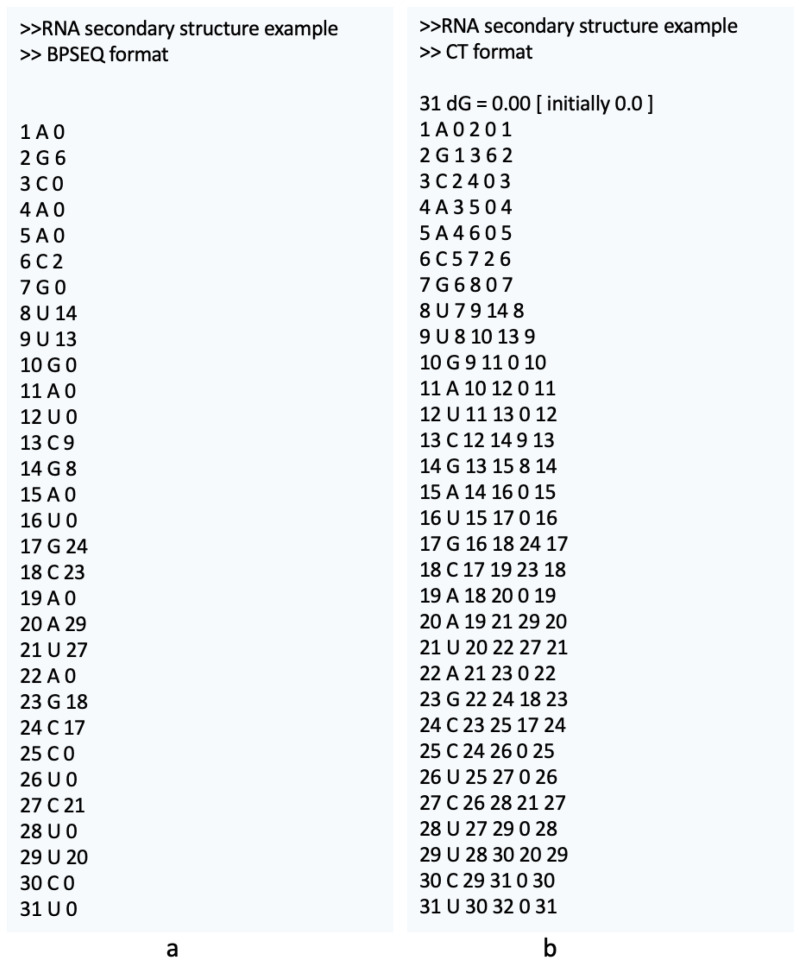
BPSEQ (**a**) and CT (**b**) representations of the RNA secondary structure from Figure 1.

Figure 5a and Figure 5b show the RNA secondary structures from Figure 1 in the BPSEQ and CT formats, respectively.

The (extended) dot-bracket format [31] consists of two lines: the first line lists the nucleotide sequence (from 5’ to 3’), and the second line describes the secondary structure using dots (for unpaired nucleotides) and various types of brackets such as (, ), [, ], {, }, <, >, or uppercase/lowercase letters to represent paired nucleotides. Non-parenthesis brackets and uppercase/lowercase notations are used to indicate pseudoknots. In the presence of pseudoknots, TARNAS employs the first-come-first-served algorithm proposed in [32] to manage their representation.

AAS [9] is a format similar to dot-bracket, but presents base pairs as a list of coordinate pairs $\left(i_{1},j_{1}\right);\left(i_{2},j_{2}\right);\ldots ;\left(i_{n},j_{n}\right)$. Each pair corresponds to the position of the paired nucleotides. The nucleotide sequence is optional in this format.

FASTA is a widely used text format for representing nucleotide or protein sequences. It consists of a sequence identifier (or header line) that begins with a > symbol, followed by a description of the sequence. This is followed by one or more lines of sequence data, typically consisting of letters that represent nucleotides (A, T, C, and G for DNA or RNA) or amino acids for proteins using IUPAC codes [33].

**Figure 6:**
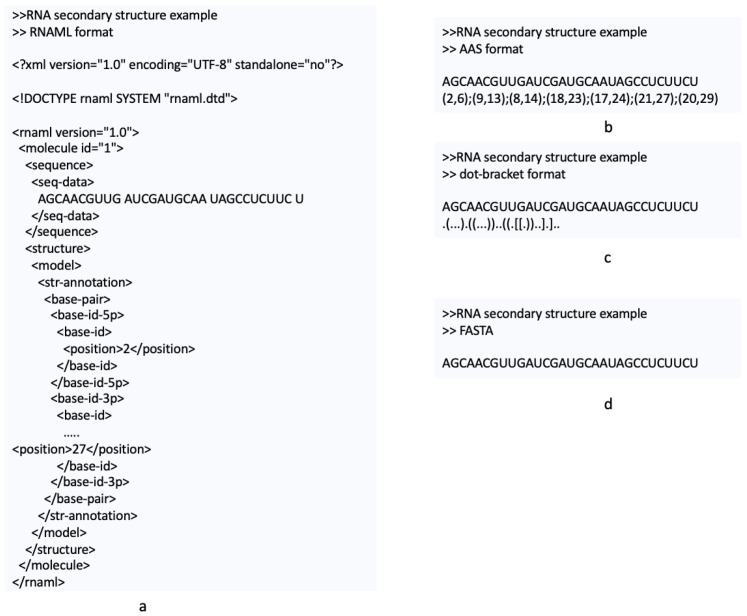
RNAML (**a**), AAS (**b**), dot-bracket (**c**), and FASTA (**d**) representations of the RNA secondary structure from Figure 1.

Figure 6a, Figure 6b and Figure 6c depict the RNA secondary structure from Figure 1 in dot-bracket, AAS, and FASTA formats, respectively.

**Figure 7:**
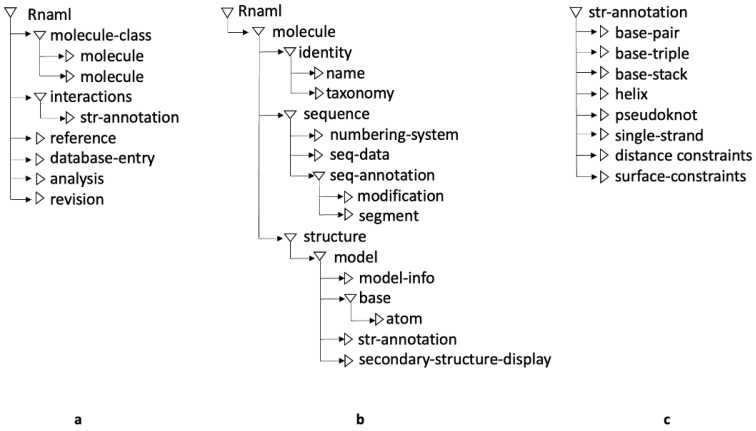
Schematic view of key RNAML markup elements. (**a**) Top-level RNAML elements. (**b**) Elements within the molecule description. (**c**) Elements used in tertiary structure annotation.

RNAML [34,35] is an XML-based format that describes RNA molecules, interactions, and relevant reference information. Figure 6a illustrates the RNA secondary structure from Figure 1 represented in RNAML. In line with the XML design principles, RNAML data are organized hierarchically using markup elements (see Figure 7). Each RNA molecule is described by an identity element that specifies its molecular name and additional details. Structural details are contained in the molecular element (Figure 7b), while higher-level components such as base pairs, stacking, helices, and pseudoknots are included in the str-annotation element (Figure 7c). Several RNAML elements are optional. Unlike the other formats, which are parsed using ANTLR4 [18,36], RNAML is processed using the W3C Document Object Model (DOM) API [19], which enables structured and hierarchical parsing of its content.

**Figure 8:**
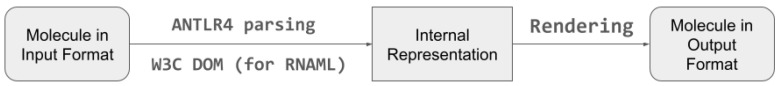
TARNAS workflow for translation. Each molecule file is parsed using either ANTLR4 or the W3C DOM, producing an internal representation of the secondary structure. This internal structure is then rendered in the requested output format.

The workflow implemented in TARNAS for format translation is depicted in Figure 8.

### 3.2. RNA Secondary Structure Abstractions

TARNAS allows users to derive three levels of abstraction for RNA secondary structures: core, core plus, and shape. These can be computed for any input molecule using a dedicated option and are returned in dot-bracket format. The abstractions are designed to reduce structural complexity while preserving features relevant to comparative and functional analysis.

The shape abstraction is derived from the arc diagram of the RNA molecule [17]. The process to obtain the shape removes all unpaired nucleotides and collapses sets of nested arcs or parallel arcs into a single representative arc. Two arcs, $\left(i_{1},j_{1}\right)$ and $\left(i_{2},j_{2}\right)$, are parallel if $i_{1}<i_{2}$ and $j_{2}>j_{1}$, with no vertices between $i_{1}$ and $i_{2}$ or between $j_{2}$ and $j_{1}$. Figure 2a shows the shape abstraction of the RNA structure represented in Figure 1.

Core and core plus [15] are derived using a similar process. The core abstraction first removes unpaired nucleotides and then collapses parallel arcs, while the core plus abstraction performs these steps in reverse. Figure 2b and Figure 2c shows the core and core plus abstraction of the RNA structure represented in Figure 1, respectively.

## 4. Conclusions

This study introduced TARNAS, a Java-based application that supports manipulating and transforming RNA secondary structure data. The tool supports conversion among formats, such as BPSEQ, CT, dot-bracket, AAS, FASTA, and RNAML. In addition to format translation, TARNAS provides functionality for abstracting RNA structures into three higher-level representations: core, core plus, and shape. The tool also offers options for generating a statistics file about the structure, including or excluding the header and retaining or discarding non-canonical base pairs when an RNAML input is provided. TARNAS is a standalone desktop application featuring GUI and CLI interfaces and a web application. The desktop version supports batch processing, enabling users to handle large numbers of RNA molecules efficiently.

In future studies, we plan to enhance TARNAS by incorporating functionalities for deriving RNA secondary structures directly from 3D molecular data, leveraging established tools such as 3DNA/DSSR [37] and RNAView [25]. We also intend to support multi-strand RNA structures, such as those found in viral genomes or RNA–RNA interaction complexes. This will involve extending simple formats like dot-bracket, CT, and BPSEQ, which typically do not distinguish whether base pairs occur within a single strand or between different strands.

In addition, we aim to compute further quantitative features, including pseudoknot order and the occurrence of key structural elements such as hairpins, helices, and loops. To support structural interpretation and improve usability, we plan to integrate visualization tools such as PseudoViewer [38], R-chie [39], VARNA [40], forna [41], and R2DT [42] for generating graphical representations of RNA secondary structures.

Finally, we plan to introduce a feature that will allow users to manually edit RNA secondary structures, enabling direct modification of base pairs or abstractions prior to analysis.

## Figures and Tables

**Table 1:** Compatibility between formats. A ✓ indicates that the translation is possible, while a × indicates that it is not. The “no seq.” versions of the dot-bracket and AAS formats contain only structural information, without the primary sequence. Formats that lack sequence data can be converted only into other structure-only formats, while formats containing only the primary sequence (such as FASTA) cannot be converted into structural formats.

	Dot-Bracket	Dot-Bracket	RNAML	CT	BPSEQ	AAS	AAS	FASTA
	No Seq						No Seq	
dot-bracket	−	×	×	×	×	×	✓	×
no seq								
dot-bracket	✓	−	✓	✓	✓	✓	✓	✓
RNAML	✓	✓	−	✓	✓	✓	✓	✓
CT	✓	✓	✓	−	✓	✓	✓	✓
BPSEQ	✓	✓	✓	✓	−	✓	✓	✓
AAS	✓	✓	✓	✓	✓	−	✓	✓
AAS no seq	✓	×	×	×	×	×	−	×
FASTA	×	×	×	×	×	×	×	−

**Table 2:** Distribution of shape motifs in the dataset.

Group	Shape	Number	Percentage
1s	([)]	717	49.1%
2s	Null Shape	667	45.6%
3s	([)([)]]	41	2.8%
4s	Other Shapes	35	2.5%

**Table 3:** Distribution of core motifs in the dataset.

Group	Core	Number	Percentage
1c	((([)])())	445	30.48%
2c	(()())	301	20.62%
3c	(((([)])())())	161	11.03%
4c	(()(()()))	115	7.88%
5c	((()())(()()))	107	7.33%
6c	((()())())	100	6.85%
7c	([[)((([)])())](()())]	31	2.12%
8c	Other Cores	200	13.70%

**Table 4:** Distribution of core plus motifs in the dataset.

Group	Core Plus	Number	Percentage
1cp	(((((([)]))))(()))	202	13.84%
2cp	(((((([)])())))(()))	102	6.99%
3cp	(((((([)]))))((())))	85	5.82%
4cp	(((((()))))(()))	62	4.25%
5cp	(((((()))))((())))	47	3.22%
6cp	(((((((()))))))((()(()))))	41	2.81%
7cp	((((((())))))((())))	34	2.33%
8cp	([[[)(((([)])()))]](()(()))]	31	2.12%
9cp	((((((()))())))(()(())))	30	2.05%
10cp	Other CorePlus	826	56.58%

**Table 5:** Statistics of the dataset. A represents the number of adenine nucleotides in the sequence and C represents the number of cytosine nucleotides, G is the number of guanine nucleotides, and U is the number of uracil nucleotides. GC is the number of bonds between G and C (in any order), AU is the number of bonds between A and U, and GU is the number of bonds between G and U. We considered only canonical Watson–Crick–Franklin base pairs and GU wobble pairs.

	Length	Bonds	A	C	G	U	GC	AU	GU
Mean	118.90	37.12	24.64	34.56	38.55	21.14	24.00	6.20	4.61
St. Dev.	4.05	3.72	3.32	4.06	4.52	4.53	4.22	3.52	1.25

## Data Availability

Data are available at [21], and code is available at https://github.com/bdslab/TARNAS (accessed on 14 May 2025) [20]. The web version of TARNAS is available at https://bdslab.unicam.it/tarnas/ (accessed on 14 May 2025).

## References

[1] Leontis N.B., Westhof E. (2001). Geometric nomenclature and classification of RNA base pairs. RNA.

[2] Shapiro B.A., Zhang K. (1990). Comparing multiple RNA secondary structures using tree comparisons. Bioinformatics.

[3] Chiu J.K.H., Chen Y.P.P. (2015). Pairwise RNA secondary structure alignment with conserved stem pattern. Bioinformatics.

[4] Hochsmann M., Voss B., Giegerich R. (2004). Pure multiple RNA secondary structure alignments: A progressive profile approach. IEEE/ACM Trans. Comput. Biol. Bioinform..

[5] Hochsmann M., Toller T., Giegerich R., Kurtz S. (2003). Local similarity in RNA secondary structures. Proceedings of the 2003 IEEE Bioinformatics Conference—CSB2003.

[6] Allali J., Sagot M.F. (2008). A multiple layer model to compare RNA secondary structures. Softw. Pract. Exp..

[7] Ouangraoua A., Ferraro P., Tichit L., Dulucq S. (2007). Local similarity between quotiented ordered trees. J. Discret. Algorithm.

[8] Blin G., Denise A., Dulucq S., Herrbach C., Touzet H. (2010). Alignments of RNA structures. IEEE/ACM Trans. Comput. Biol. Bioinform..

[9] Quadrini M., Tesei L., Merelli E. (2020). ASPRAlign: A tool for the alignment of RNA secondary structures with arbitrary pseudoknots. Bioinformatics.

[10] Zok T., Badura J., Swat S., Figurski K., Popenda M., Antczak M. (2020). New models and algorithms for RNA pseudoknot order assignment. Int. J. Appl. Math. Comput. Sci..

[11] Gan H.H., Fera D., Zorn J., Shiffeldrim N., Tang M., Laserson U., Kim N., Schlick T. (1987). RAG: RNA-As-Graphs database—Concepts, analysis, and features. Nutr. Health.

[12] Antczak M., Zok T., Popenda M., Lukasiak P., Adamiak R.W., Blazewicz J., Szachniuk M. (2014). RNApdbee—A webserver to derive secondary structures from pdb files of knotted and unknotted RNAs. Nucleic Acids Res..

[13] Zok T., Antczak M., Zurkowski M., Popenda M., Blazewicz J., Adamiak R.W., Szachniuk M. (2018). RNApdbee 2.0: Multifunctional tool for RNA structure annotation. Nucleic Acids Res..

[14] Quadrini M., Tesei L., Merelli E. (2022). Automatic generation of pseudoknotted RNAs taxonomy. BMC Bioinform..

[15] Quadrini M., Hierro Chancari P., Rosati P., Tesei L., Cerulo L., Napolitano F., Bardozzo F., Cheng L., Occhipinti A., Pagnotta S. (2025). TARNAS, a TrAnslator for RNA Secondary Structure Formats. Proceedings of the 19th International Meeting, CIBB 2024.

[16] Bon M., Vernizzi G., Orland H., Zee A. (2008). Topological classification of RNA structures. J. Mol. Biol..

[17] Huang F.W., Reidys C.M. (2015). Shapes of topological RNA structures. Math. Biosci..

[18] Parr T. (2013). The Definitive ANTLR 4 Reference.

[19] Flanagan D. (2011). JavaScript: The Definitive Guide.

[20] Quadrini M., Hierro Canchari P., Rosati P., Tesei L. (2025). Release of TARNAS 1.0.0, a software tool for RNA secondary structure abstraction and format translation. Zenodo.

[21] Quadrini M., Canchari P.H., Rosati P., Tesei L. (2025). TARNAS—A Tool for Translating and Abstracting RNA Secondary Structures—Software Documentation and Dataset. Zenodo.

[22] Walls C. (2016). Spring Boot in Action.

[23] Tilkov S., Vinoski S. (2010). Node.js: Using JavaScript to build high-performance network programs. IEEE Internet Comput..

[24] Freeman A. (2022). Pro Angular: Build Powerful and Dynamic Web Apps.

[25] Yang H., Jossinet F., Leontis N., Chen L., Westbrook J., Berman H., Westhof E. (2003). Tools for the automatic identification and classification of RNA base pairs. Nucleic Acids Res..

[26] Burley S.K., Berman H.M., Kleywegt G.J., Markley J.L., Nakamura H., Velankar S. (2017). Protein Data Bank (PDB): The Single Global Macromolecular Structure Archive. Protein Crystallography: Methods and Protocols.

[27] Reidys C.M., Huang F.W., Andersen J.E., Penner R.C., Stadler P.F., Nebel M.E. (2011). Topology and prediction of RNA pseudoknots. Bioinformatics.

[28] Cannone J.J., Subramanian S., Schnare M.N., Collett J.R., D’Souza L.M., Du Y., Feng B., Lin N., Madabusi L.V., Müller K.M. (2002). The comparative RNA web (CRW) site: An online database of comparative sequence and structure information for ribosomal, intron, and other RNAs. BMC Bioinform..

[29] Johnson A.D. (2010). An extended IUPAC nomenclature code for polymorphic nucleic acids. Bioinformatics.

[30] Mathews D.H., Sabina J., Zuker M., Turner D.H. (1999). Expanded sequence dependence of thermodynamic parameters improves prediction of RNA secondary structure. J. Mol. Biol..

[31] Hofacker I.L., Fontana W., Stadler P.F., Bonhoeffer S., Tacker M., Schuster P. (1994). Fast folding and comparison of RNA secondary structures. Monatshefte Chem./Chem. Mon..

[32] Antczak M., Popenda M., Zok T., Zurkowski M., Adamiak R.W., Szachniuk M. (2018). New algorithms to represent complex pseudoknotted RNA structures in dot-bracket notation. Bioinformatics.

[33] Pearson W.R., Lipman D.J. (1988). Improved tools for biological sequence comparison. Proc. Natl. Acad. Sci. USA.

[34] Masuya H., Griffiths-Jones S., Bateman A., Quang T.T., Gaudin N., Lowe T.M. (2006). RNAML: A Standard Syntax for Exchanging RNA Information. Bioinformatics.

[35] Waugh A., Gendron P., Altman R., Brown J.W., Case D., Gautheret D., Harvey S.C., Leontis N., Westbrook J., Westhof E. (2002). RNAML: A standard syntax for exchanging RNA information. RNA.

[36] Parr T. ANTLR Website. https://www.antlr.org/.

[37] Lu X.J., Olson W.K. (2003). 3DNA: A software package for the analysis, rebuilding and visualization of three-dimensional nucleic acid structures. Nucleic Acids Res..

[38] Byun Y., Han K. (2009). PseudoViewer3: Generating planar drawings of large-scale RNA structures with pseudoknots. Bioinformatics.

[39] Lai D., Proctor J.R., Zhu J.Y.A., Meyer I.M. (2012). R-CHIE: A web server and R package for visualizing RNA secondary structures. Nucleic Acids Res..

[40] Darty K., Denise A., Ponty Y. (2009). VARNA: Interactive drawing and editing of the RNA secondary structure. Bioinformatics.

[41] Kerpedjiev P., Hammer S., Hofacker I.L. (2015). Forna (force-directed RNA): Simple and effective online RNA secondary structure diagrams. Bioinformatics.

[42] McCann H., Meade C.D., Williams L.D., Petrov A.S. (2021). R2DT: A comprehensive platform for visualizing RNA secondary structure. Nat. Commun..

